# Prevalence and risk factors of preconception anemia: A community based cross sectional study of rural women of reproductive age in northeastern Tanzania

**DOI:** 10.1371/journal.pone.0208413

**Published:** 2018-12-18

**Authors:** Omari A. Msemo, Ib C. Bygbjerg, Sofie L. Møller, Birgitte B. Nielsen, Lars Ødum, Kathrine Perslev, John P. A. Lusingu, Reginald A. Kavishe, Daniel T. R. Minja, Christentze Schmiegelow

**Affiliations:** 1 National Institute for Medical Research, Tanga Centre, Tanga, Tanzania; 2 Section of Global Health, Department of Public Health, University of Copenhagen, Copenhagen, Denmark; 3 Department of Obstetrics and Gynecology, Aarhus University Hospital, Aarhus, Denmark; 4 Department of Clinical Biochemistry, Roskilde Hospital, Rokslide, Denmark; 5 Centre for Medical Parasitology, Department of Immunology and Microbiology, University of Copenhagen, Copenhagen, Denmark; 6 Kilimanjaro Christian Medical University College, Moshi, Tanzania; London School of Hygiene and Tropical Medicine, UNITED KINGDOM

## Abstract

**Background:**

Anemia is a major public health problem that adversely affects pregnancy outcomes. The prevalence of anemia among pregnant women before conception is not well known in Tanzania. The aim of this study was to determine the prevalence, types, and risk factors of preconception anemia in women of reproductive age from a rural Tanzanian setting.

**Methods:**

Trained field workers visited households to identify all female residents aged 18–40 years and invited them to the nearby health facility for screening and enrolment into this study. Baseline samples were collected to measure hemoglobin levels, serum ferritin, vitamin B_12_, folate, C-reactive protein, alanine amino-transferase, the presence of malaria, HIV, and soil transmitted helminth infections. Anthropometric and socio-economic data were recorded alongside with clinical information of participants. Logistic regression analysis was used to determine the adjusted odds ratios (AOR) for the factors associated with preconception anemia.

**Findings:**

Of 1248 women enrolled before conception, 36.7% (95% confidence interval (CI) 34.1–39.4) had anemia (hemoglobin <12 g/dL) and 37.6% (95% CI 34.9–40.4) had iron deficiency. For more than half of the anemic cases, iron deficiency was also diagnosed (58.8%, 95% CI 54.2–63.3). Anemia was independently associated with increased age (AOR 1.05, 95% CI 1.03–1.07), malaria infection at enrolment (AOR 2.21, 95% CI 1.37–3.58), inflammation (AOR 1.77, 95% CI 1.21–2.60) and iron deficiency (AOR 4.68, 95% CI 3.55–6.17). The odds of anemia were reduced among women with increased mid-upper arm circumference (AOR 0.90, 95% CI 0.84–0.96).

**Conclusion:**

Anemia among women of reproductive age before conception was prevalent in this rural setting. Increased age, iron deficiency, malaria infection and inflammation were significant risk factors associated with preconception anemia, whereas increased mid-upper arm circumference was protective against anemia. Interventions to ensure adequate iron levels as well as malaria control before conception are needed to prevent anemia before and during pregnancy and improve birth outcomes in this setting.

**Trial registration:**

NCT02191683.

## Introduction

Anemia, a condition characterized by insufficient hemogolobin (Hb) concentration to meet the oxygen demand of the tissue, affects nearly one-quarter of the world’s population [1,2]. Distinct Hb cut-offs to define anemia varies by age, sex, altitude, smoking, and pregnancy status and are available in guidelines put forward by the World Health Organization (WHO) [3]. For non-pregnant women anemia is defined as Hb<12 g/dL [3]. Whereas, during pregnancy the cut-offs varies by gestation ageas a result of increased blood volume and plasma expansion, and is <11g/dL during the first trimester, <10.5 g/dL during the second trimester and <11 g/dL during the third trimester. In sub-Saharan Africa (SSA) anemia is prevalent among women of reproductive age and young children [1,2,4]. In year 2011, it was estimated that 496 million (29%) non-pregnant and 32 million (38%) pregnant women had anemia globally [2], and the World Health Organization (WHO) set a target to reduce by 50% anemia among women of reproductive age by year 2025 [4].

The etiology of anemia is multifactorial with the prevalence and causes varying considerably in different areas of the world by population group, region, residence (urban or rural), socioeconomic status (SES), and general environmental factors [1,5]. Iron is a key component of Hb, and its deficiency is estimated to be responsible for 50% of all anemia cases in SSA [2,6–8]. Iron demands are high during pregnancy and increased parity and gravidity and short interpregnancy interval can therefore substantially reduce maternal iron reserves [9]. Additional risk factors include other micronutrient deficiencies (folate, vitamins B_12_ and A), malaria, soil transmitted helminths (STH), chronic infections like HIV, genetic disorders including sickle cell anemia, glucose-6-phosphate dehydrogenase (G6PD) deficiency and, α-thalassemias as well as other inflammatory conditions [10].

The consequences of anemia and ID vary depending on the severity, the population groups and living conditions but includes reduced work capacity in adults and neurocognitive impairment in young children [11]. Furthermore, it is well established that anemia during pregnancy is associated with intrauterine growth retardation, preterm delivery [12,13], low birthweight [12–15], and perinatal mortality [16].To address this, pregnant women in Tanzania are prescribed iron and folate supplements but compliance is poor due to side effects [17]. Vitamin B_12_ deficiency causes megaloblastic anemia and infant vitamin B_12_ deficiency, but it is not included in the micronutrients supplementation program because of insufficient data supporting its implementation [18].

Although anemia during pregnancy has been associated with adverse pregnancy outcomes, the proportion of women who are already anemic or had depleted iron stores just before conception has not been well investigated in SSA. Therefore, the aim of this study was to determine the prevalence, types and risk factors associated with preconception anemia among women of reproductive age in a rural setting of northeastern Tanzania.

## Materials and methods

### Ethical statement

The study received ethical approval from the Medical Research Coordinating Committee of the National Institute for Medical Research (reference number NIMR/HQ/R.8a/Vol. IX/1717).Written informed consent or thumbprint (for illiterate women) was obtained prior to enrolment. All study procedures were performed according to good clinical and laboratory practices and the Declaration of Helsinki [19].

### Study design and setting

This cross sectional study was conducted as part of a community-based epidemiological study entitled “Foetal exposure and epidemiological transition: the role of anemia in early life for non-communicable diseases in later life” (FOETALforNCD) fromJuly 2014 to December 2016 in Korogwe and Handeni districts, Tanga region, Tanzania. The aim of theFOETALforNCD project was toevaluate fetal growth alterations, placental development, and newborn susceptibility to non-communicable diseases in later life, following exposure to maternal anemia before and during pregnancy. The study population composed of women of the reproductive age. The analyses presented here utilized baseline data from women enrolled before they became pregnant. Inclusion into this study was based on their likelihood to conceive during the study period. To be included, women had to be aged 18–40 years, not be using modern contraceptive methods (except condom), or not be sub-fertile (defined as failure to conceive for two or more consecutive years for women who were trying to become pregnant), or not be pregnant at the time of enrolment (negative urine pregnancy test, HCG Vista Care Company, Shandong China), or not have a baby less than nine months old and live in an accessible area, and be willing to receive antenatal care and deliver at Korogwe District Hospital.

### Participant identification and recruitment

Different stakeholders including village leaders, health care providers, opinion makers as well as community members were sensitized about the study goals and aims through village and health facility meetings prior to the implementation of the FOETALforNCD study. The primary means of identifying and recruiting eligible women was through contact at the household level within each village. Trained field workers made door-to-door visits to each household to explain the study, enumerate all women of reproductive age, and issue invitation cards for them to visit the nearby health facility for screening and enrolment. Other awareness and recruitment strategies included regular home visits by trained field workers (to identify new women moving into established households) and screening women as they sought other health care services. Eligible women were informed that after conception, the intention was to follow them throughout pregnancy until delivery.

Upon conception transabdominal ultrasound (5–2 MHz abdominal probe, Sonosite TITAN and Sonosite Turbo, US High resolution, Sonosite, Bothell, WA, USA) was used to estimate gestational age (GA). Gestational age estimation was based on measurement of crown rump length in the first trimester [20] and head circumference in the second trimester [21].

**Fig 1:**
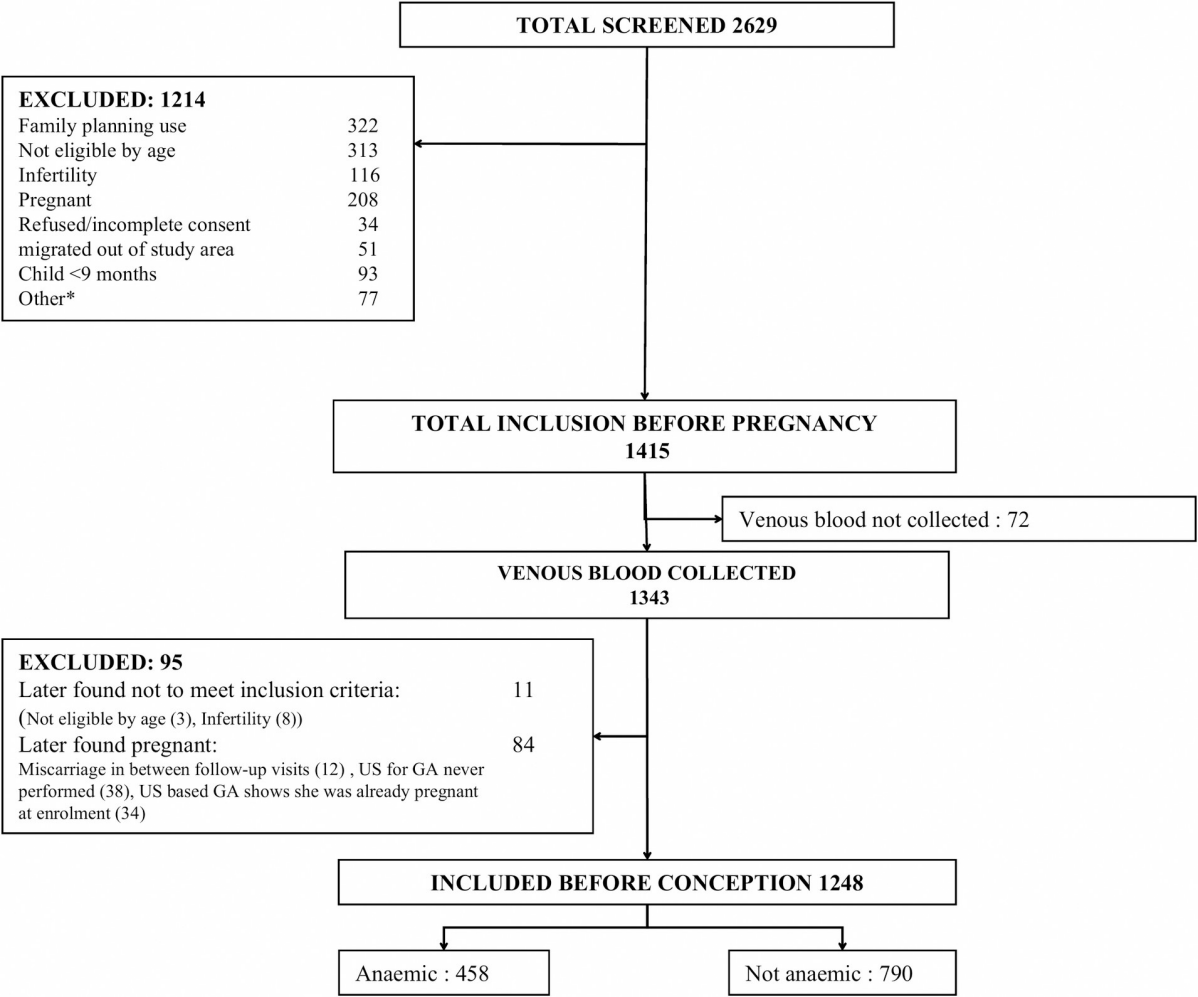
Flow chart of participants in FOETALforNCDprepregnancy cohort.

From July 2014 to December 2015, 2629 women were screened for eligibility for inclusion into the FOETALforNCD study and 1415 were enrolled. Of the 1214 exclusions, 313 (25.8%) were not eligible by age, 322 (26.5%) were still using modern family planning methods, 116 (9.6%) were sub-fertile, 208 (16.6%) were already pregnant, 34 (2.8%) refused, 51(4.2%) migrated out of study area and 93 (7.7%) had a child <9 months old, while 77 (6.3%) were excluded due to other reasons. Of the 1415women included, 72 were later excluded because venous blood was not collected, and 11 did not fulfil the inclusion criteria. Furthermore, 84 women who conceived during the follow up were excluded because; 34 were already pregnant at enrolment based on the ultrasound estimated GA and 50 had a miscarriage before the GA could be ascertained, leaving 1248 women for the present analysis (Fig 1).

### Data collection and tools

Socio-demographic data including age, educational level, marital status, and economic (household size, house ownership and type of roofing materials, main source of drinking water and its ownership (private or public),type of toilet facility) and lifestyle factors (smoking, alcohol and tea consumption)were collected using a structured questionnaire. Previous medical histories, including gynecological and obstetric details, were documented. In order to define SES, a principal component analysis was applied and the variables which showed relevant contribution (greater than 10%) to the combined SES score were regarded as the ones which sufficiently described the SES of a woman [22]. Variables included in the final principal component analysis were educational level, occupation, type of house ownership, roofing materials, source of domestic water and its ownership as well as the type of toilet facility. The respective SES scores were categorized in tertiles as low, medium and high.

Weight (in kilograms) was measured while on barefoot and wearing light clothes (precision 0.1kg, digital weighing scales, SecaGmbh& Co. Kg, Hamburg, Germany). Height in centimeters (cm) was measured with a stadiometer (precision 1 cm) [23]. Mid-upper arm circumference (MUAC) was measured on the upper right arm at the midpoint of the acromion process and the tip of the olecranon (precision 1mm). For measurement of skinfold thickness, trained staff pinched the skin above triceps muscle group to raise a double layer of skin and the underlying adipose tissue without the muscle. The HARPENDEN skinfold caliper (BATY International, England) was then applied 1 cm above and at right angle to the pinch, and a reading in millimeters (mm) taken after a few second. Waist circumference was measured just above the iliac crest in the horizontal plane, and hip circumference was measured at the point yielding the maximum circumference over the buttocks, all using a standard measuring tape to the nearest 1mm[24].

At enrolment, 15ml of venous blood was collected in ethylenediamine tetra acetic acid coated and plain serum tubes, transported at 2° to 8°C to the NIMR Korogwe Research Laboratory and processed within two hours of collection. To avoid photo degradation during transportation, all plain tubes were wrapped in aluminium foil. Separated serum samples were stored at -80°C and later shipped in dry ice to University Hospital Sealand, Denmark for micronutrients analysis.

Hemoglobin level was measured by using Sysmex KX-21N hematological analyzer (Sysmex Corporation Kobe, Japan).According to WHO’s definition, anemia was defined as Hb<12.0 g/dL, and further categorized as mild (10.1–11.9 g/dL), moderate (8.0–10.0 g/dL) and severe (<8.0 g/dL) [3]. Microcytosis was defined as mean corpuscular volume (MCV) value <80 fL and hypochromic as mean cell hemoglobin concentration (MCHC) value <32 g/dL. Anemia was further classified as normocytic-normochromic (Hb<12 g/dL, MCV 80–100 fL and MCHC>32 g/dL), microcytic hypochromic (Hb<12 g/dL, MCV< 80 fL and MCHC<32 g/dL), megaloblastic (Hb<12g/dL, MCV≥100) or as mixed types (normocytic-hypochromic, microcytic-normochromic macrocytic-normochromic and macrocytic-hypochromic) anemia. For clinical care, Hb levels were measured using HemoCue 301 Hb analyzer (HemoCue AB, Angelholm, Sweden). Anemic women received treatments as follows: mild anemic (Hb10.1–11.9 g/dL) women with no symptoms received dietary counseling whereas women with symptoms were offered one combination tablet of 200 mg ferrous sulfate (~ 43 mg elemental iron) and 400μg folate per day (Ferrolic–LF, Laboratory and Allied LTD, Mombasa, Kenya). Moderately anemic patients with Hb 9.1–10.0g/dL received 2–3 combination tablets of iron and folic acid (Ferrolic–LFLaboratory and Allied LTD, Mombasa, Kenya) per day and monitored at each scheduled visit. Those with Hb 8.0–9.0 g/dL received a daily dose of 20 mL Hemovit multivitamin syrup (200 mg Ferrous sulfate, 0.5mg B_6_, 50 μg B_12_, 1500 μg Folic acid and 2.33mg Zinc per 5mL, Shelys Pharmaceuticals, Dar es Salaam, Tanzania) and monitored at each scheduled visit. Severely anemic (Hb<8g/dL) women were referred to Korogwe District Hospital for further evaluation.

Serum ferritin, vitamin B_12,_ folate, alanine aminotransferase (ALT) and bilirubin levels were measured using Dimension Vista 1500 biochemical analyzer (Siemens Healthcare Diagnostics, Inc, New York, USA) [25]. C-reactive protein (CRP) was measured by using Afinion AS 100 analyzer (Axis Shield PoC, AS, Oslo Norway) and inflammation defined as serum CRP >5 mg/L and/or ALT >45 U/L [10].

To account for elevated serum ferritin due to sub-clinical infection and other inflammatory conditions, three approaches were applied to define ID and results compared. The first approach utilized arithmetic correction factor (CF)as proposed by Thurnham*et al*.[26] to adjust for the increased serum ferritin levels due to inflammation. In this approach CF of 0.67 was applied only for samples that had evidence of inflammation (CRP>5 mg/L), and a cut-off of <15 μg/L was then applied to the adjusted ferritin levels to define ID. If CRP was not available, serum ferritin was coded as missing. In the second approach, a higher ferritin-cutoff (<30μg/L) was applied to the subset of individuals with inflammation (CRP >5 mg/L) to define ID, as proposed by the WHO [27]. In this approach ID was defined as serum ferritin <15μg/L (no inflammation) or 30μg/L (inflammation present). The third approach utilized the higher serum ferritin cutoff (<30μg/L) if CRP>5mg/L and/or ALT>45 U/L which is considered as a sign of liver disease [10]. In the core analyses presented here, ID anemia was defined as Hb<12g/dL in the presence of ID based on Thurnham approach. Vitamin B_12_ deficiency was defined as serum cobalamin <150 pmol/L, and folate deficiency as serum folate<10 nmol/L without adjusting for inflammation [28].

Malaria was diagnosed using malaria rapid diagnostic test (mRDT) kit, ParaHIT (span diagnostics, Gujarat, India) or CareStart Malaria Pf (HPR2), ACCESS BIO, New Jersey, USA) according to manufacturer instructions. In addition, thick and thin blood films were prepared for the detection and quantification of parasitemia. Malaria patients received oral artemether-lumefantrine, (Lumartem 20mg/120mg (Cipla Ltd, Patalganga, India), quinine or artesunate injections according to Tanzanian standard treatment guideline. Human immunodeficiency virus infection was tested by using DetermineHIV-1/2 test kit (Alere ltd, Stockport, UK) and seropositive cases were confirmed using Unigold test kit (Trinity Biotech Plc, Wicklow, Ireland) according to the manufacturers’ instructions. Newly diagnosed HIV patients were referred to the nearby care and treatment clinics for long-term care. Considering low prevalence of STH infestations in north eastern Tanzania [29], stool samples were collected from a subgroup of 434 women at the time of enrolment and preserved in 10% neutral buffered formalin solution. Formol-ether concentration technique was used to detect presence of STH infestations [30]. All confirmed (on stool samples)or clinical suspected cases of STH infestation received a single dose of albendazole (400mg) or mebendazole (500mg) tablets according to the existing Tanzanian standard treatment guideline.

#### Statistical analysis

Microsoft Access software 2007 (Microsoft corporation, Redmond’s, USA) was used for data entry and validation. Stata version 13 (StataCorp, Lake Way drive, College station, USA) software was used for statistical analyses. Continuous variables were visually inspected for normality using histograms and described using mean and standard deviation if normally distributed or median (interquartile range—IQR) for skewed data. Univariate analysis was done using Student’s t-test or Mann-Whitney test for continuous parametric and non-parametric variables, and Chi-square (χ^2^) or Fisher's exact test for categorical variables. Factors associated with preconception anemia were determined using logistic regression analysis and expressed as unadjusted odds ratio (OR) and adjusted odds ratios (AOR). All variables with *P*-value <0.20 in the univariate analysis were entered into the multivariate models [31]. Using a stepwise backward elimination approach final models including variables with a *P*-value <0.10 were obtained. A *P*-value of *<*0.05 was considered statistically significant. Due to missing data on HIV infections in 350 (28.8%) women and considering HIV infection being an important confounding factor, two different models with and without adjusting for HIV infection were generated and compared.

Finally, in order to illustrate the association between a risk factor and anemia, trendline figures were generated for each continuous risk factor found to be statistically significant or borderline significant in the multivariate model.

## Results

The baseline characteristics of the women enrolled in the study are shown inTable 1 below.

**Table 1:** Baseline characteristics of women before pregnancy in north eastern Tanzania.

		All		Anemic (Hb<12g/dL)		Non-anemic (Hb≥12g/dL)	
Characteristics	N	% (95% CI) /median (IQR)	n	% (95% CI) /median(IQR)	n	% (95% CI) /median (IQR)	*P*-value
**Age (yrs)**^**a**^	1233	28 (22–34)	448	29 (22–36)	785	27.8 (22–33)	0.004
**Ethnic group**^**b**^	1247		457		790		0.021
Sambaa		34.0 (31.4–36.7)		38.3(33.9–42.9)		31.5 (28.4–34.9)	
Zigua		32.5 (29.9–36.7)		32.4 (28.2–36.8)		32.5 (29.3–35.9)	
Others^**b**^		33.5 (30.9–36.2)		29.3 (25.3–33.8)		36.0 (32.7–39.4)	
Parity^**b**^	1242		456		786		0.067
0		18.4 (16.4–20.7)		18.4 (12.1–22.3)		18.4 (15.9–21.))	
1		38.7 (36.1–41.5)		35.5 (31.3–25)		40.6 (37.2–31.4)	
2		16.5 (14.5–18.7)		16.0 (12.9–19.7)		16.8 (14.3–19.6)	
3		12.4 (10.7–14.4)		12.3 (12.0–15.9)		15.0 (12.5–14.8)	
>3		13.9 (12.1–16.0)		17.5 (14.3–21.3)		11.8 (9.8–14.3)	
**House hold size (numbers)**^**a**^	1243	4.0 (3.6)	457	4 (3–6)	786	4 (3–6)	0.31
**Education level**^**b**^	1247		457		790		0.12
No education		8.1(6.7–9.8)		9.0 (6.7–12.0)		7.6 (5.9–9.7)	
Primary incomplete		11.2 (9.5–13.1)		11.4 (8.8–14.6		11.1(9.1–13.5)	
Primary school complete		63.1(60.4–65.8)		65.4 (60.9–69.7)		61.8 (58.3–65.1)	
Secondary or higher		17.6 (15.5–19.8)		14.2 (11.3–17.7)		19.5 (16.9–22.4)	
**Occupation**^**b**^	1247		457		790		0.53
Professional		3.1(2.2–4.2)		3.3 (2.0–5.4)		2.9 (1.9–4.3)	
Business		3.4 (2.6–4.6)		3.5 (2.2–5.6)		3.4 (2.4–4.9)	
Service provider		15.2 (13.3–17.3)		12.9 (10.1–16.3)		16.6 (14.1–19.3)	
Farmer		57.2 (54.4–59.9)		59.1 (54.4–63.5)		56.1 (52.6–59.5)	
House wife/student		21.1(19.9–223.4)		21.2 (17.7–25.2)		21.0 (18.3–24.0)	
**Type of house ownership**^**b**^	1242		456		786		0.21
Self/spouse built		64.3 (61.6–67.0)		66.5 (62.0–70.6)		63.1 (59.7–66.4)	
Inherited		15.7 (13.8–17.8)		16.2 (13.1–19.9)		15.4 (13.0–18.1)	
Rental		20.0 (17.8–22.3)		17.3(14.1–21.1)		21.5 (18.8–24.5)	
**Roofing materials**^**b**^	1247		457		790		0.36
Iron		74.0 (71.0–76.3)		73.3 (69.0–77.2)		74.3 (71.1–77.2)	
Tin		0.6 (0.3–1.3)		0.2 (0.03–1.5)		0.9 (0.4–1.8)	
Asbestos		1.1(0.6–1.8)		1.5 (0.7–3.2)		0.9 (0.4–1.8)	
Thatch/mixed		24.3 (22.0–26.8)		25.0 (21.2–29.1)		23.9 (21.1–27.0)	
**Source of domestic water** ^**b**^	1244		455		786		0.64
Private tap		7.7 (6.4–9.4)		8.5 (6.3–11.5)		7.2 (5.6–9.3)	
Private bore hole		0.8 (0.4–1.5)		1.1 (0.4–2.6)		0.6 (0.3–1.5)	
Public tap		53.7 (50.9–56.4)		51.6 (47.0–56.2)		54.6 (51.1–58.2)	
Public bore hole		11.5 (9.9–13.4)		11.0 (8.4–14.2)		12.0 (9.7–14.3)	
River/pool		26.3 (23.9–28.8)		27.8 (23.9–32.1)		25.6 (22.7–28.8)	
**Type of toilet facility**^**b**^	1246		456		790		0.38
Flush		25.0 (22.7–27.5)		23.9 (20.2–28.1)		25.7 (22.8–28.9)	
Pit		74.1 (71.6–76.4)		74.8 (70.6–78.6)		73.7 (70.5–76.6)	
None		0.9 (0.5–1.6)		1.3 (0.6–2.9)		0.6 (0.3–1.5)	
**Socioeconomic status**^**b**^^,^^**b**^	1235		453		782		0.25
High		33.5 (30.9–36.2)		35.5 (31.2–40.1)		32.3 (29.2–35.7)	
Medium		33.4 (30.8–36.0)		34.2 (30.0–38.7)		32.9 (29.7–36.2)	
Low		33.1 (30.5–35.8)		30.2 (26.2–34.6)		34.8 (31.5–38.2)	
**Bed net ownership (Yes)**^**b**^	1247	78.8 (76.5–81.0)	457	77.2 (72.3–80.9)	790	79.7 (76.8–82.4)	
**MUAC (cm)** ^**a**^	1242	27.9 (25.8–31)	453	27.4 (25.4–29.9)	789	28 (26–31.5)	0.0003
**Waist circumference (cm)**^**a**^	1218	80.0 (74.1–87.8)	444	79.2 (74.2–86.1)	774	80.3 (74–89)	0.06
**Hip circumference(cm)**^**a**^	1234	94.5 (89–101.2)	454	94 (88.1–99.5)	780	95 (89.4–102.1)	0.01
**Body mass index (kg/m**^**2**^**)**^**a**^	1231	22.7 (20.5–26.3)	449	22.1 (20.2–25.2)	782	23.0 (20.7–26.7)	0.036
**Body mass index categories**							
Underweight	1231	8.0 (6.6–9.7)		9.4 (7.0–12.4)		7.2 (5.7–9.3)	
Normal weight		60.5 (57.8–63.2)		63.9 (59.4–68.2)		58.9 (55.1–62.0)	
Overweight		20.2 (18.1–22.6)		18.0 (14.7–21.9)		21.5 (18.7–24.5)	
Obesity		11.2 (9.6–13.1)		8.7 (6.4–11.7)		12.7 (10.5–15.3)	
**Previous use of hormone contraceptives**^**b**^	1248	71.5 (68.9–73.9)	458	32.5 (63.0–77.6)	790	26.2 (23.2–29.4)	0.017
**Length of menstrual periods**^**b**^	1217	4 (3–5)	446	4 (3–5)	771	4 (3–5)	0.104
**Length of menstrual cycle (days)**^**b**^	1162		428		734		0.144
Normal (25–35)		85.7 (83.6–87.6)		84.1 (80.3–87.3)		86.7 (84.0–88.9)	
Short (<25)		2.9 (2.1–4.1)		2.3 (1.2–4.3)		3.3 (2.2–4.8)	
Long (≥35)		11.4 (9.7–13.3)		13.6 (10.6–17.1)		10.0 (8.1–12.5)	
**Self-reported malaria last 2 months**^**b**^	1234	22.7 (20.4–25.1)	452	26.1(22.3–30.4)	782	20.7 (18.0–23.7)	0.029
**Malaria at enrolment**^**b**^	1244	8.1 (6.7–9.8)	456	10.3 (7.8–13.4)	788	6.9 (5.3–8.8)	0.032
**HIV seropositive**^**e**^	952	5.7 (4.4–7.3)	344	9.0 (6.4–12.6)	608	3.8 (2.5–5.6)	0.004
**Intestinal helminths infestations**^**b**^	434	3.0 (1.7–5.1)	147	1.4 (0.3–5.4)	287	4.9 (2.1–6.8)	0.64
**Inflammation (CRP>5mg/L)**^**b**^	1229	15.2 (13.3–17.3)	448	18.5 (15.2–22.4)	781	13.3 (11.1–15.9)	0.014
**Hemoglobin (g/dL)(mean±se)**	1248	12.2±0.04	458	10.7±0.06	790	13.1±0.03	<0.001
**RBC morphological types**^**b**^							
Normocytic-normochromic	1248	56.3 (53.6–59.3)	458	32.8 (28.6–37.2)	790	70.0 (66.7–73.1)	<0.001
Microcytic-hypochromic	1248	17.5 (15.5–19.8)	458	38.2 (33.9–42.8)	790	5.6 (4.2–7.4)	
Microcytic-normochromic	1248	19.9 (17.7–22.2)	458	19.0 (15.6–22.9)	790	20.4 (17.7–23.3)	
Normocytic-hypochromic	1248	5.9 (4.7–7.4)	458	9.8 (7.4–12.9)	790	3.7 (2.6–5.2)	
Megaloblastic	1248	0.3 (0.1–0.8)	458	0.2 (0.03–1.5)	790	0.4 (0.1–1.4)	
**Micronutrient status**							
s-ferritin (μg/L)^**a**^	1245	22.3 (10.1–39.7)	457	11.3 (5.4–28.4)	788	27.3 (15.2–43.8)	<0.001
Iron deficiency ^**b**^	1226	37.6 (34.9–40.4)	447	58.8 (54.2–63.3)	779	25.4 (22.5–28.6)	<0.001
s-Folate (nmol/L)^**a**^	1194	30.8 (21–39.3)	448	28.8 (19.7–37.8)	746	32.2 (22.4–40.4)	0.0003
Folate deficiency^**b**^	1194	2.1 (1.4–2.9)	448	3.1 (1.8–5.2)	746	1.5 (0.8–2.6)	0.043
s-Vitamin B_12_(pmol/L)^**a**^	1245	465.2 (343–613.4)	457	455.8 (332.7–591.7)	788	470.9 (347.7–626.8)	0.07
Vitamin B12 deficiency^**b**^	1245	0.6 (0.3–1.2)	457	0.4 (0.1–1.7)	788	0.6 (0.2–1.5)	0.65
**Time from enrolment to conception (days)**^**f**^	259	87 (38–157)	91	85 (32–143)	168	91 (40–160)	0.48
**Adverse pregnancy outcomes**^**g**^	249	34.1 (28.5–40.3)	86	34.9 (25.4–45.7)	163	33.7 (26.8–41.4)	0.86

^a^Median (IQR)

^b^ Percentage (95% CI)

^c^Other ethnic group comprised of 56 minority groups that constituted <10% of the total study population per group.

^d^Socio-economic status was determined using principal components analysis based on woman education level, occupation, main source of domestic water, type of house ownership, roofing materials and type of toilet facility. Low scores for SES were given for no education/partial primary education, occupation as housewife/farmer, pond/river, rental housing, thatch roofing, or having no toilet.

^e^ HIV infection was tested only for women who consented after voluntary counseling. Iron deficiency was based on Thurnham*et al*. approach [26]. There were no mixed microcytic and macrocytic RBC morphological types.

^f^ During the study period 259 women conceived and had gestational age estimated using ultrasound and data during pregnancy available. Women conceiving after the end of the study did not provide data for these analyses.

^g^ Adverse pregnancy outcomes include miscarriage, low birthweight, prematurity, small for gestational age (<10^th^percentile on the STOPPAM reference chart)[32] or stillbirth.

**Abbreviations:** Hb: Hemoglobin; MUAC: Mid-upper arm circumference; RBC: Red blood cell; CRP: C—reactive protein

The median (IQR) age of the women was 28.2 (IQR = 22–34) years. Few women were underweight (8.0%), while 60.5%, 20.2% and 11.2%, were normal weight, overweight and obese, respectively. Majority (71.5%) had previously used at least one type of hormonal contraceptive. Malaria infection was detected in 8.1% (95% CI 6.7–9.8) of the women while 5.7% (95% CI 4.4–7.3), 3.0% (95% CI 1.7–5.1) and 15.2% (95% CI 13.3–17.3) had STH infestations, HIV and inflammation, respectively.

The mean±seHb level was 12.2±0.04 g/dL and 36.7% (95% CI 34.1–39) women had anemia (Table 1). Severe, moderate, and mild anemia were found in 1.8%, 95% CI 1.8–2.7), 16.1% (95% CI 14.2–18.3 and 18.8% (95% CI 16.8–21.1) of women, respectively (data not shown). Comparing anemic and non-anemic women, there was a statistically significant difference in age, ethnicity, MUAC, waist and hip circumference, BMI, and self-reported or malaria infection at the time of enrolment (Table 1). Overweight (BMI of 25-<30kg/m^2^) and obesity (BMI≥30 kg/m^2^) were observed in 20.2% (95% CI 18.1–22.6%) and 11.2% (95% CI 9.6–13.1) of the women, respectively. The coexistence of overweight and obesity with anemia was found in 81/449 (18.0%) and 39/449(8.6%) of women, respectively (Table 1) while79/451 (17.5%) and 42/451 (10.0%) of iron deficient women had concurrent overweight and obesity, respectively (data not shown).

When using the internal CF approach by Thurnham*et al*., 37.6% (95% CI 34.9–40.4) women had ID. Using the WHO definition (serum ferritin <15 μg/L or <30 μg/L in the presence of inflammation), 39.2% (95% CI 36.5–41.9) were classified as having ID. After further adjustment to account for liver disease (serum ferritin <15μg/L or <30μg/L in the presence of inflammation (CRP >5 mg/Land ALT >45 U/L), 40.2% (95% CI 37.5–43.0) women were classified as having ID. Of the 458 women with anemia 58.8% (95% CI 54.2–63.3), 3.1% (95% CI 1.8–5.1) and 0.4% (95% CI 0.1–1.7) had concurrent iron, folate and vitamin B_12_ deficiencies, respectively (Table 1). Based on Thurnham approach, among 461 women with ID, only 2.2% (95% CI 1.2–4.1) had combined iron and folate deficiency and 0.4% (95% CI 0.1–1.7) had combined iron and vitamin B_12_ deficiencies, while none had combined folate and Vitamin B_12_ deficiency (data not shown).Finally, concurrent ID with malaria, HIV and inflammation were found in 2.8% (95% CI 1.7–4.8), 4.7% (95% CI 3.0–7.5), and 11.7% (95% CI 9.1–15.1), respectively (data not shown).

The majority of the women (56.3%, 95% CI 53.6–59.3) had normocytic-normochromic RBC morphology, while 17.5% (95% CI 15.5–19.8), 19.9% (95% CI 17.7–22.2), 5.9% (95% CI 4.7–7.4) and 0.2% (95% CI 0.1–0.8) had microcytic-hypochromic, microcytic-normochromic, normocytic-hypochromic and megaloblastic RBC morphology, respectively. However, for the anemic women, only 32.8% (95% CI 28.6–37.2) had normocytic-normochromic while 38.2% (95% CI 33.9–42.8), 19.0% (95% CI 15.6–22.9), 9.8% (95% CI 7.4–12.9) and 0.2% (95% CI 0.03–1.5) had microcytic-hypochromic, macrocytic normochromic, normocytic-hypochromic and megaloblastic RBC morphology, respectively (Table 1). Interestingly, among the ID anemic women (Hb<12g/dL and serum ferritin <15μg/L), only 48.7% (95% CI 42.6–54.7) had microcytic-hypochromic RBC morphological pattern, whereas 25.5% (95% CI 20.5–31.1), 20.6% (95% CI 17.1–24.6) and 6.5% (95% CI 4.5–9.2) had normocytic-normochromic, microcytic-normochromic and normocytic-hypochromic RBC morphological types, respectively (data not shown).

The results of the univariate and multivariate logistic regression analysis with all variables entered into the model except HIV infection are shown in Table 2 below.

**Table 2:** Risk factors associated with preconception anemia among women in rural north eastern Tanzania (N = 1125) ^a^.

	Unadjusted		Adjusted^b^	
Characteristics	OR (95% CI)	*P*-value	OR (95% CI)	*P*- value
**Age(years)**	1.03 (1.01–1.04)	0.003	1.05 (1.03–1.07)	<0.001
**Ethnic group**				
Sambaa	Ref.		Ref.	
Zigua	0.82 (0.62–1.08)	0.163	0.82 (0.59–1.13)	0.231
Others^c^	0.67 (0.51–0.89)	<0.001	0.63 (0.45–0.87)	0.005
**Parity**				
0	Ref.		Ref.	
1	0.75 (0.51–1.09)	132	0.80 (0.49–1.31)	0.382
2	1.04 (0.71–1.53)	0.828	1.16 (0.67–1.99)	0.597
3	0.96 (0.64–1.41)	0.817	1.01 (0.55–1.86)	0.981
≥4	1.24 (0.88–1.76)	0.226	1.13 (0.59–2.16)	0.703
**MUAC (cm)**	0.95 (0.92–0.98)	<0.001	0.90 (0.84–0.96)	0.002
**Waist circumference (cm)**	0.99 (0.98–0.99)	0.03	1.02 (0.98–1.06)	0.698
**Hip circumference (cm)**	0.98 (0.97–0.99)	0.007	1.02 (0.99–1.05)	0.060
**Body Mass Index**	0.96 (0.93–0.98)	<0.001	0.97 (0.89–1.07)	0.589
**Previous use of hormonal contraceptives**	0.74 (0.57–0.95)	0.017	0.74 (0.54–1.00)	0.053
**Length of menstrual periods**	1.09 (1.01–1.17)	0.035	1.05 (0.96–1.16)	0.269
**Length of menstrual cycles (days)**				
Normal (25–35)	Ref		Ref	
Short (<25)	1.38 (0.96–2.00)	0.082	1.26 (0.81–1.95)	0.298
Long(≥35)	0.73 (0.35–1.56)	0.423	0.82 (0.34–2.01	0.668
**Self-reported malaria last two months**	1.35 (1.03–1.77)	0.035	1.34 (0.98–1.84)	0.068
**Malaria at enrolment**	1.56 (1.04–2.35)	0.033	2.21 (1.37–3.58)	0.001
**Inflammation**	1.48 (1.08–2.03)	0.015	1.77 (1.21–2.60)	0.003
**Iron deficiency**	4.19 (3.27–5.37)	<0.001	4.68 (3.55–6.17)	<0.001
**Folate deficiency**	2.15 (0.87–5.29)	0.059	2.15 (0.87–5.29)	0.097

^a^Final model consisted of 1125 observations due to some missing values

^b^Only variables with p<0.1 were retained in the final multivariate analysis model.

^c^Other ethnic group comprised of 56 minority groups that constituted <10% of the total study population per group. Iron deficiency was based on Thurnham*et al*. approach [26].

**Abbreviation:** MUAC: Mid-upper arm circumference.

In the univariate analysis, women from the minority tribes, increased MUAC, waist and hip circumferences, BMI as well as previous use of hormonal contraceptives, were statistically significantly associated with reduced risk of anemia. Increasing age, length of menstrual periods, malaria infection before or at the time of enrolment, inflammation and ID were significantly associated with increased risk of anemia (Table 2). Skinfold thickness showed similar associations with risk of anemia as MUAC but data was missing on 25% of the women and were therefore not included in further analyses (data not shown).

In the multivariate logistic regression analysis, women from the minority tribes (adjusted OR (AOR) 0.63, 95% CI 0.45–0.87) and increased MUAC (AOR 0.90, 95% CI 0.84–0.96) remained statistically significantly associated with reduced odds of having anemia while previous use of hormonal contraceptives was marginally protective against anemia (AOR 0.74, 95% CI 0.54–1.00). Increased age (AOR 1.05, 95% CI 1.03–1.07), malaria infection at the time of enrolment (AOR) 2.21, 95% CI 1.37–3.58), inflammation (AOR 1.77, 95%, CI 1.21–2.60), and ID (AOR 4.68, 95% CI 3.55–6.17 were all statistically significantly associated with increased risk of anemia (Table 2). A model including HIV was also generated (S1 Table). The AORs for anemia were similar for increased age, ethnicity, and malaria infection at the time of enrolment, inflammation and ID as in the model without HIV. Human immunodeficiency virus infection led to a three-fold increased risk of anemia (AOR2.57, 95%CI 1.37–4.81). Increased MUAC was no longer significantly associated with increased risk of anemia after adjusting for HIV infection (S1 Table).

**Fig 2:**
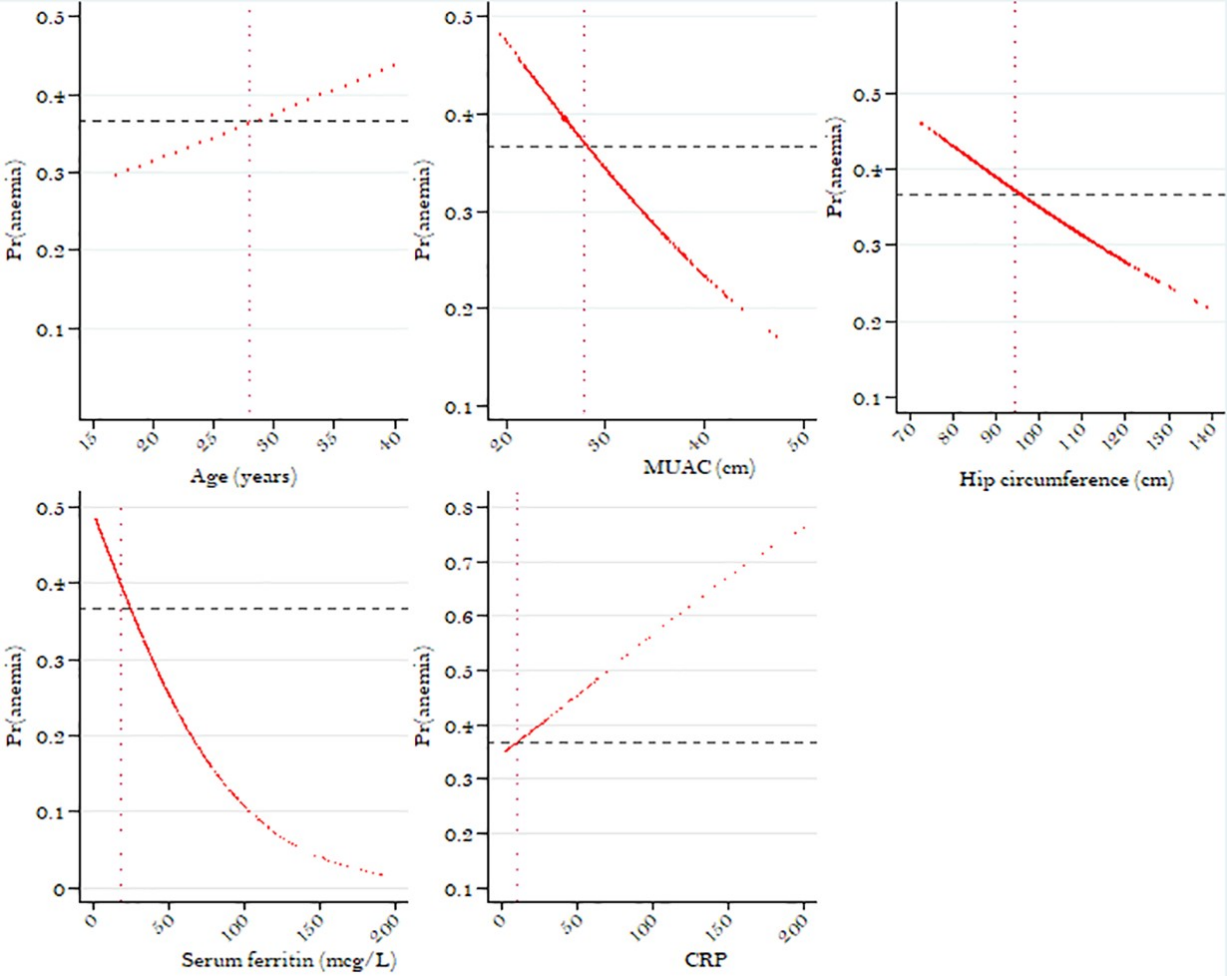
Trendline graphs of proportions of anemia by age, MUAC, hip circumference, s-ferritin and CRP among non-pregnant women in Korogwe.

The risk of anemia gradually and in linear manner decreased with increasing MUAC and hip circumference but it increased with increasing age and CRP level. However, with a MUAC<28cm, hip circumference<95 cm, age >28yearsand CRP >10mg/L the prevalence of anemia was higher than the average prevalence of 36.7%. Finally, the risk of anemia steadily decreased with increasing ferritin levels up to 100μg/L and then flattened out, but the prevelence of anemia was above the average only at ferritin concentrations below 20μg/L (Fig 2).

## Discussion

The present study assessed the prevalenceand risk factors of preconceptionanemia in a cohort of rural Tanzanian women who were likely to conceive. In this study, a significant proportion of women had anemia and ID before conception. The prevalence of anemia in this study was similar to other community based studies among non pregnant women of reproductive age in Tanzania [7], Ethiopia [6] and Sierra Leone [8], which reported prevalence of anemia of 39.5%,30.4% and 44.8%, respectively,but with a wide variation in the prevalence of ID. In this study all women who were currently using modern contraceptives were excluded but the observed prevalence of anemia and ID were comparable to what Haile *et al*.[7] reported among non-pregnant Tanzanian women of reproductive age including hormonal contraceptive users. Since all women in this study were not using modern contraceptive methods and hence were likely to conceive, over one third are at risk of having anemia and/or suboptimal iron levels at the time of conception without interventions. The ID and anemia may further deteriorate during pregnancy in response to the increased physiologic demands of gestation, thereby increasing the risk of adverse pregnancy outcomes.

The risk of anemia decreased with increasing MUAC. Previous studies have also showed a negative correlation between anemia and MUAC [33,34]. In low resource settings, MUAC has been shown to correctly identify undernourished or anemic women [35,36], but there is no consensus on appropriate anthropometric cut-off points for risk prediction.Despite increasing adiposity being protective against anemia, pregnant women who are overweight or obese may face increased risk of numerous other complications, including gestational diabetes, pre-eclampsia, pregnancy-induced hypertension, stillbirths and preterm births [37]. Moreover, clustering of obesity and ID or anemia within the same individual poses a great challenge for public health interventions. In the current study, we found that nearly one third of overweight/obese women had concurrent anemia or ID. Furthermore, not until a MUAC >28cm was the prevalence of anemia lower than the average of 36.7% indicating that some women are still anemic despite having a normal weight or only slight overweight. Therefore, specific interventions aimed at preventing anemia before conception should target all women of reproductive age, irrespective of their nutrition status.

Iron deficiency was common among both non-anemic as well as anemic women, but ID was associated with an almost five-fold increased risk of anemia. This finding is comparable to the recent study by Haile *et al*. [7] and underscores that adequate iron stores among women of the reproductive age is important and might be ensured by routine provision of iron and folic acid supplementation to all menstruating women, as recommended by the WHO [38].

Iron deficiency was characterized based on both RBC morphology (MCV and MCHC) and serum ferritin and compared, but a large proportion of the women with true ID would have been misclassified as not having ID based on RBC morphological patterns alone. This is not surprising as up to 40% of pure ID anemia may present with normocytic-normochromic pattern [39]. Moreover, the presence of microcytosis does not necessarily imply ID and can be present due to other causes such as thalassemias[40], or anemia of chronic disease [41]. Likewise, dimorphic anemia can occur in partially treated ID [41]. Serum ferritin is an acute phase reactant and can be increased if inflammation is present [42]. We applied the CF [26] to get a more precise estimate of ID in all women who had inflammation. Interestingly, the prevalence of ID using the CF did not differ significantly as compared to the two other used approaches (higher ferritin cut-off and or/ adjusting for high ALT) [42]. This was in contrast to a recent study by Namaste *et al*.[27] who reported a substantial underestimation of the burden of ID when only a higher ferritin cut-off approach was used as compared to adjusted approaches. One possible explanation for this difference could be the smaller sample size in our study compared to Namaste *et al*. study [27]. Haile *et al*. [7] excluded all malaria patients and used serum transferrin receptor (sTfR) as a marker of ID. However, sTfR can also be affected by acute inflammation [43,44], and recently adjustment of sTfR using internal CF has been proposed [44]. Likewise, exclusion of all women with malaria would have introduced a selection bias.

A much higher prevalence of ID was observed as compared to Wirth *et al*. who reported a prevalence of ID of 8.3% among rural non-pregnant women of reproductive age in Sierra Leone despite a high prevalence of anemia (44.3%) [8]. The difference could be explained by a higher prevalence of malaria in Sierra Leone (23%) as compared to 8.1% in our study. Likewise, a recent meta-analysis has shown that the contribution of ID to the overall burden of anemia in women of reproductive age is much lower in regions with high burden of malaria and inflammation [5].

Although both malaria and HIV prevalence was below 10%, both were statistically significant risk factors for anemia and public health strategies should target these conditions. This should include screening and treatment to reduce the consequences for those already infected as well as preventive measures such as bed nets and condom usage in order to reduce the risk of infection. We also observe a trend towards a reduced risk of anemia for women who had previously used hormonal contraceptives. Similar, to our study, Haile *et al*., found that hormonal contraceptive use was significantly associated with a reduced risk of anemia and ID among non-pregnant women in Tanzania [7]. This might partially be due to reduced menstrual bleeding, as well as indicative of a higher socio-economic status and hence better health status among hormonal contraceptives users. For example, a recent report has shown that that 35% of women in the wealthiest households used modern contraceptive methods as compared to 20% among women from low income households [45].

The prevalence of STH infestations was very low and among the anemic women, none had infestations. This finding is similar to a previous study in this region which showed very low prevalence of STH (0.3%) among children in an urban settings [29]. The area participates in annual mass drug administration for control of lymphatic filariasis using ivermectin and albendazole as well as school deworming program. These programs may have shown additional impact on reduction of STH infestations.

Dietary iron absorption from the human gut is dependent on physiological requirements, but may be restricted by the quantity or bioavailability [46]. Whether IDin this population was primarily the result of dietary insufficiency or problems with absorption was beyond the scope of this paper. Understanding these patterns could provide important information about the relationship between food consumption and the risk of ID and anemia before conception in this setting. However, previous studies from a neighboring district of Muheza has shown that 73% of total iron intake came from staple food mainly cereals, fats and nuts and green leafy vegetables [46].In addition to low bioavailability, cereal staples contain iron absorption inhibitors, particularly tannins and phytates[46]. Therefore, high prevalence of ID without concurrent folate deficiency in our study could signify poor bioavailability rather than dietary insufficiency.

The Tanzanian government has strengthened different interventions to reduce the burden of anemia during pregnancy, including iron and folic acid supplementation, deworming, intermittent preventive treatment for malaria (IPTp) with sulfadoxine pyrimethamine as well as scaling up the coverage and usage of insecticide treated bed nets[45]. Furthermore, the Tanzanian National Multisectoral Nutrition Action Plan (2016–2021) aims to reduce the proportion of women of reproductive age with anemia from 45% to 33% through increased uptake of iron and folate supplements during pregnancy, increased acess to micronutrient powders from 10%-35% and increased proportion of iron fortified flour produced in Tanzania from 36%-50% [47]. However, despite anticipated benefits, the initiative has been compromised by high prevalence of pre-existing anemia and ID and short period of supplementation [45].Daily supplementation with 30–60 mg of elemental iron taken for three consecutive months in a year, is recommended by the WHO as a public health intervention to prevent anemia and ID for menstruatng women and adolescent girls living in settings where the prevalence of anemia 40% or higheror intermittent iron and folic acid supplementation in settings where prevelence of anemia is 20–40% [38]. However, despite its proven benefit, and recommendation by the WHO[38],preconception iron and folic acid supplementation has not been included in the current Tanzanian National Multisectora Nutrition Action Plan [47]. It is therefore worrisome that many anemic women willreceive these supplements only after conception and after having booked for antenatal care.

The strengths of the study include a large number of socio-demographic, economic, and clinical variables collected and stringent data monitoring system to ensure the quality of collected data. Furthermore, we could be rather certain that the cohort was truly preconception. For women who conceived during follow up, 85% had GA estimation in the first trimester. Pregnant women were excluded from analysis if the ultrasound examination demonstrated that they were already pregnant before conception or the GA was uncertain.

On the other hand, this study had several limitations. Firstly, the design of the main study was to identify women who would potentially conceive during the study period, especially women who were not using modern contraceptives. Women who were still using traditional contraceptive methods were included and their behavior and SES could be different from women who were actively trying to become pregnant. Secondly, serum ferritin level, which is also an acute phase protein, was used to detect ID. Serum ferritin levels were adjusted to account for the effect of inflammation by using internal CF and exclusion of all women without CRP measurement but this might not have corrected the entire effect. Further adjustment by using alfa1 acid glycoprotein in addition to CRP as marker of inflammation as well as using internal regression approach have also been proposed [27]. An alternative was to use sTfR, which is less affected by inflammation, but it was not available in this study. Thirdly, our study utilized serum sample to measure folate and B_12_ levels. The sensitivity of serum folate and vitamin B_12_ measurements for the diagnosis of clinically significant deficiency is uncertain because of the lack of a gold standard diagnostic method [48]. However, this may have had little effect on our estimate of the proportion of women with folate or vitamin B_12_ since previous studies have shown these deficiencies are responsible for a small proportion of anemia among women and children in SSA [7,8]. Finally, the present study did not determine the burden of vitamin A deficiency is also a risk factor of anemia.

## Conclusion

The prevalence of preconception anemia and ID in this study population was high, while vitamin B_12_ and folate deficiencies were rare. Iron deficiency, malaria and HIV infections as well as inflammation were significantly associated with preconception anemia. This signifies that many women will enter pregnancy with depleted iron stores and/or anemia putting themselves and their growing fetus at risk. Therefore, concerted efforts, including intermittent iron and folic acid supplementation and nutrition counseling to improve iron status before conception for optimal fetal growth and newborn short and long-term health should also be considered in this population.

## Supporting information

S1 Table Risk factors of preconception anemia (including HIV infection) among rural women in north- eastern Tanzania. **Note**:^a^Only variables with p<0.1 were retained in the final multivariate analysis model ^b^Other ethnic group comprised of 56 minority groups that constituted <10% of the total study population per group. **Abbreviations:** MUAC: Mid upper arm circumference; RBC: Red blood cells.
(DOC)

S1 Dataset Preconception anemia in Korogwe (deidentified dataset).dta. (DTA)

## References

[1] Balarajan Y, Ramakrishnan U, Ozaltin E, Shankar AH,Subramanian SV Anaemia in low-income and middle-income countries. Lancet 2011; 378: 2123–2135. 10.1016/S0140-6736(10)62304-5 21813172

[2] Stevens GA, Finucane MM, De-Regil LM, Paciorek CJ, Flaxman SR, Branca F et al Global, regional, and national trends in haemoglobin concentration and prevalence of total and severe anaemia in children and pregnant and non-pregnant women for 1995–2011: a systematic analysis of population-representative data. Lancet Glob.Health 2013; 1: e16–e25. 10.1016/S2214-109X(13)70001-9 PMC4547326 25103581

[3] World HealthOrganization. Haemoglobin concentrations for the diagnosis of anaemia and assessment of severity. Vitamin and Mineral Nutrition Information System. (2011).Available from www.who.int/vmnis/indicators/haemoglobin/en/. 9-2-2018.

[4] World Health Organization. Global nutrition targets 2025: anaemia policy brief (2014).Available from www.who.int/iris/handle/10665/148556. 25-1-2018

[5] Petry N, Olofin I, Hurrell RF, Boy E, Wirth JP, Moursi M et al The Proportion of Anemia Associated with Iron Deficiency in Low, Medium, and High Human Development Index Countries: A Systematic Analysis of National Surveys. Nutrients 2016; 8. 10.3390/nu8110693 PMC5133080 27827838

[6] Haidar J. Prevalence of anaemia, deficiencies of iron and folic acid and their determinants in Ethiopian women. J.Health Popul.Nutr. 2010; 28: 359–368. 20824979 10.3329/jhpn.v28i4.6042 PMC2965327

[7] Haile ZT, Kingori C, Teweldeberhan AK, Chavan B. The relationship between history of hormonal contraceptive use and iron status among women in Tanzania: A population-based study. Sexual & Reproductive Healthcare 2017; 13: 97–102. 28844365 10.1016/j.srhc.2017.07.003

[8] Wirth JP, Rohner F, Woodruff BA, Chiwile F, Yankson H, Koroma AS et al Anemia, Micronutrient Deficiencies, and Malaria in Children and Women in Sierra Leone Prior to the Ebola Outbreak—Findings of a Cross-Sectional Study. PLoS One 2016; 11: e0155031 10.1371/journal.pone.0155031 27163254 PMC4862671

[9] Low MS, Speedy J, Styles CE, De-Regil LM, Pasricha SR. Daily iron supplementation for improving anaemia, iron status and health in menstruating women. Cochrane.Database.Syst.Rev. 2016; 4: CD009747 10.1002/14651858.CD009747.pub2 27087396 PMC10182438

[10] Suchdev PS, Williams AM, Mei Z, Flores-Ayala R, Pasricha SR, Rogers LM et al Assessment of iron status in settings of inflammation: challenges and potential approaches. Am.J.Clin.Nutr. 2017; 106: 1626S–1633S. 10.3945/ajcn.117.155937 29070567 PMC5701714

[11] Nyaradi A, Li J, Hickling S, Foster J, Oddy WH. The role of nutrition in children's neurocognitive development, from pregnancy through childhood. Frontiers in human neuroscience 2013; 7. 10.3389/fnhum.2013.00097 PMC3607807 23532379

[12] Dewey KG, Oaks BM. U-shaped curve for risk associated with maternal hemoglobin, iron status, or iron supplementation. Am J Clin Nutr 2017; 106: 1694S–1702S. 10.3945/ajcn.117.156075 29070565 PMC5701708

[13] Kozuki N, Lee AC, Katz J. Moderate to severe, but not mild, maternal anemia is associated with increased risk of small-for-gestational-age outcomes. J.Nutr 2012; 142: 358–362. 10.3945/jn.111.149237 22190028

[14] Ribot B, Aranda N, Viteri F, Hernandez-Martinez C, Canals J, Arija V. Depleted iron stores without anaemia early in pregnancy carries increased risk of lower birthweight even when supplemented daily with moderate iron. Hum.Reprod. 2012; 27: 1260–1266. 10.1093/humrep/des026 22357769

[15] Ronnenberg AG, Wood RJ, Wang X, Xing H, Chen C, Chen D et al Preconception hemoglobin and ferritin concentrations are associated with pregnancy outcome in a prospective cohort of Chinese women. J.Nutr. 2004; 134: 2586–2591. 10.1093/jn/134.10.2586 15465752

[16] Wrottesley SV, Lamper C, Pisa PT. Review of the importance of nutrition during the first 1000 days: maternal nutritional status and its associations with fetal growth and birth, neonatal and infant outcomes among African women. J.Dev.Orig.Health Dis. 2016; 7: 144–162. 10.1017/S2040174415001439 26279311

[17] Ogundipe O, Hoyo C, Ostbye T, Oneko O, Manongi R, Lie RT et al Factors associated with prenatal folic acid and iron supplementation among 21,889 pregnant women in Northern Tanzania: a cross-sectional hospital-based study. BMC Public Health 2012; 12: 481 10.1186/1471-2458-12-481 22734580 PMC3438116

[18] Bellows AL, Smith ER, Muhihi A, Briegleb C, Noor RA, Mshamu S et al Micronutrient Deficiencies among Breastfeeding Infants in Tanzania. Nutrients 2017; 9: 1258. 10.3390/nu9111258 PMC5707730 29149073

[19] Dixon JR The international conference on harmonization good clinical practice guideline. Quality Assurance: Good Practice, Regulation, and Law 1999; 6: 65–74. 10.1080/105294199277860 10386329

[20] Papageorghiou AT, Kennedy SH, Salomon LJ, Ohuma EO, Cheikh IL, Barros FC et al International standards for early fetal size and pregnancy dating based on ultrasound measurement of crown-rump length in the first trimester of pregnancy. Ultrasound Obstet.Gynecol. 2014; 44: 641–648. 10.1002/uog.13448 25044000 PMC4286014

[21] Papageorghiou AT, Kemp B, Stones W, Ohuma EO, Kennedy SH, Purwar M et al Ultrasound-based gestational-age estimation in late pregnancy. Ultrasound Obstet.Gynecol. 2016; 48: 719–726. 10.1002/uog.15894 26924421 PMC6680349

[22] Vyas S, Kumaranayake L Constructing socio-economic status indices: how to use principal components analysis. Health policy and planning 2006; 21: 459–468. 10.1093/heapol/czl029 17030551

[23] Schmiegelow C, Minja D, Oesterholt M, Pehrson C, Suhrs HE, Bostrom S et al Malaria and fetal growth alterations in the 3(rd) trimester of pregnancy: a longitudinal ultrasound study. PLoS One 2013; 8: e53794 10.1371/journal.pone.0053794 23326508 PMC3543265

[24] World Health Organization. Waist circumference and waist-hip ratio: Report of a WHO expert consultation, Geneva, (2008). Available from www.who.int/nutrition/publications/obesity/WHO_report_waistcircumference_and_waisthip_ratio/en/. 16-12-2017.

[25] Bruneel A, Dehoux M, Barnier A, Boutten A External evaluation of the Dimension Vista 1500-« intelligent lab system. Journal of clinical laboratory analysis 2012; 26: 384–397. 10.1002/jcla.21539 23001985 PMC6807579

[26] Thurnham DI, McCabe LD, Haldar S, Wieringa FT, Northrop-Clewes CA, McCabe GP. Adjusting plasma ferritin concentrations to remove the effects of subclinical inflammation in the assessment of iron deficiency: a meta-analysis. Am.J.Clin.Nutr. 2010; 92: 546–555. 10.3945/ajcn.2010.29284 20610634

[27] Namaste SM, Rohner F, Huang J, Bhushan NL, Flores-Ayala R, Kupka R et al Adjusting ferritin concentrations for inflammation: Biomarkers Reflecting Inflammation and Nutritional Determinants of Anemia (BRINDA) project. Am.J.Clin.Nutr. 2017; 106: 359S–371S. 10.3945/ajcn.116.141762 28615259 PMC5490647

[28] De Benoist B Conclusions of a WHO Technical Consultation on folate and vitamin B12 deficiencies. Food and Nutrition Bulletin 2008; 29: S238–S244. 10.1177/15648265080292S129 18709899

[29] Mwakitalu ME, Malecela MN, Mosha FW,Simonsen PE Urban schistosomiasis and soil transmitted helminthiases in young school children in Dar es Salaam and Tanga, Tanzania, after a decade of anthelminthic intervention. Acta tropica 2014; 133: 35–41. 10.1016/j.actatropica.2014.01.012 24495630

[30] Suwansaksri J, Nithiuthai S, Wiwanitkit V, Soogarun S, Palatho P. The formol-ether concentration technique for intestinal parasites: comparing 0.1 N sodium hydroxide with normal saline preparations. Southeast Asian J.Trop.Med.Public Health 2002; 33 Suppl 3: 97–98. 12971485

[31] Douglas G.Altman Practical Statistics for Medical Research Chapman and Hall/CRC 1991; 349.

[32] Schmiegelow C, Scheike T, Oesterholt M, Minja D, Pehrson C, Magistrado P et al Development of a fetal weight chart using serial trans-abdominal ultrasound in an East African population: a longitudinal observational study. PLoS One 2012; 7: e44773 10.1371/journal.pone.0044773 23028617 PMC3448622

[33] Zillmer K, Pokharel A, Spielman K, Kershaw M, Ayele K, Kidane Y et al Predictors of anemia in pregnant women residing in rural areas of the Oromiya region of Ethiopia. BMC Nutrition 2017; 3: 65– 10.1186/s40795-017-0166-y PMC7050732 32153845

[34] Ghosh S, Trevino JA, Davis D, Shrestha R, Bhattarai A, Anusree KC et al Factors associated with anemia in pregnant women in Banke, Nepal. The FASEB Journal 2017; 31: 788–32.

[35] Nguyen P, Ramakrishnan U, Katz B, Gonzalez-Casanova I, Lowe AE, Nguyen H et al Mid-upper-arm and calf circumferences are useful predictors of underweight in women of reproductive age in northern Vietnam. Food and Nutrition Bulletin 2014; 35: 301–311. 10.1177/156482651403500303 25902590

[36] Hinderaker SG, Olsen BE, Lie RT, Bergsjo PB, Gasheka P, Bondevik GT et al Anemia in pregnancy in rural Tanzania: associations with micronutrients status and infections. Eur.J.Clin.Nutr. 2002; 56: 192–199. 10.1038/sj.ejcn.1601300 11960293

[37] Khan MN, Rahman MM, Shariff AA, Rahman MM, Rahman MS, Rahman MA. Maternal undernutrition and excessive body weight and risk of birth and health outcomes. Archives of Public Health 2017; 75: 12 10.1186/s13690-017-0181-0 28174626 PMC5291969

[38] World Health Organization. Guideline: daily iron supplementation in adult women and adolescent girls (2016).World Health Organization. Available at http://apps.who.int/iris/handle/10665/204761 31-5-2018 27195351

[39] Bermejo F, Garcia-Lopez S. A guide to diagnosis of iron deficiency and iron deficiency anemia in digestive diseases. World J.Gastroenterol. 2009; 15: 4638–4643. 10.3748/wjg.15.4638 19787826 PMC2754511

[40] Zimmermann MB Methods to assess iron and iodine status. Br J Nutr 2008; 06/01: S2–S9. 10.1017/S000711450800679X 18598585

[41] Ford J Red blood cell morphology. International Journal of Laboratory Hematology 2013; 35: 351–357. 10.1111/ijlh.12082 23480230

[42] World Health Organization. Serum ferritin concentrations for the assessment of iron status and iron deficiency in populations (2011).World Health Organization. Available at www.who.int/vmnis/indicators/serum_ferritin.pdf. 19-1-2018.

[43] Engle-Stone R, Nankap M, Ndjebayi AO, Erhardt JG, Brown KH. Plasma ferritin and soluble transferrin receptor concentrations and body iron stores identify similar risk factors for iron deficiency but result in different estimates of the national prevalence of iron deficiency and iron-deficiency anemia among women and children in Cameroon. J.Nutr 2013; 143: 369–377. 10.3945/jn.112.167775 23343673

[44] Rohner F, Namaste SM, Larson LM, Addo OY, Mei Z, Suchdev PS et al Adjusting soluble transferrin receptor concentrations for inflammation: Biomarkers Reflecting Inflammation and Nutritional Determinants of Anemia (BRINDA) project. Am.J.Clin.Nutr. 2017; 106: 372S–382S. 10.3945/ajcn.116.142232 28615256 PMC5490651

[45] National Bureu of Statistics. Tanzania Demographic and Health Survey and Malaria indicator survey 2015–2016 (2016). Available at www.nbs.go.tz 5-6-2018

[46] Ecker O, Weinberger K, Qaim M. Patterns and determinants of dietary micronutrient deficiencies in rural areas of East Africa. African Journal of Agricultural and Resource Economics 2010; 4: 175–194.

[47] The United Republic of Tanzania:Prime minister's office. National Multisectoral Nutrition Action Plan (NMNP)-From Evidence to Policy to Action (2016). Available at https://www.unicef.org/tanzania/NMNAP_2016-21_Final_version_for_printing_12082017.pdf. 11-11-2018.

[48] Snow CF Laboratory diagnosis of vitamin b12 and folate deficiency: A guide for the primary care physician. Archives of Internal Medicine 1999; 159: 1289–1298. 10386505 10.1001/archinte.159.12.1289

