# Supplementary material for: Prevalence and risk factors of preconception anemia: A community based cross sectional study of rural women of reproductive age in northeastern Tanzania
Source: PLoS One. 2018 Dec 18;13(12):e0208413. doi: 10.1371/journal.pone.0208413 (PMC6298689; doi:10.1371/journal.pone.0208413)
Supplement: S1 Table — Note:aOnly variables with p<0.1 were retained in the final multivariate analysis model bOther ethnic group comprised of 56 minority groups that constituted <10% of the total study population per group. Abbreviations: MUAC: Mid upper arm circumference; RBC: Red blood cells. (DOC) [file pone.0208413.s001.doc]

**Table S1 Risk factors of preconception anemia (including HIV infection) among rural women in north eastern Tanzania (n=905**)

|  | **Unadjusted** | | **Adjusted^a^** | |
| --- | --- | --- | --- | --- |
| **Characteristics** | **OR (95% CI)** | ***P*-value** | **OR (95% CI)** | ***P*-value** |
| **Age(years)** | 1.04 (1.02-1.06) | 0.001 | 1.03 (1.01-1.06 ) | 0.005 |
| **Ethnic group** |  |  |  |  |
| Sambaa | Ref. |  | Ref. |  |
| Zigua | 0.71 (0.51-0.99) | 0.04 | 0.87 (0.61-1.24) | 0.438 |
| Others^b^ | 0.52 (0.37-0.74) | <0.001 | 0.68 (0.48-0.99) | 0.049 |
| **Parity** |  |  |  |  |
| 0 | Ref. |  | Ref. |  |
| 1 | 0.75 ( (0.47-1.20) | 0.239 | 0.74 (0.42-1.29) | 0.291 |
| 2 | 1.09 (0.69-1.74) | 0.702 | 1.05 (0.57-1.93) | 0.885 |
| 3 | 0.99 (0.62-1.58) | 0.969 | 0.92 (0.47-1.82) | 0.815 |
| ≥4 | 1.34 (0.89-2.04) | 0.164 | 0.93 (0.46-1.87) | 0.840 |
| **MUAC (cm)** | 0.96 (0.93-0.99) | 0.019 | 0.96 (0.93-1.00) | 0.068 |
| **Hip circumference(cm)** | 0.99 (0.98-1.00) | 0.059 | 1.02 (0.98-1.05) | 0.354 |
| **Body Mass Index** | 0.96 (0.94-0.99) | 0.015 | 0.99 (0.91-1.08) | 0.835 |
| **Previous used hormonal contraceptives** | 0.75 (0.55-1.01) | 0.056 | 0.81 (0.56-1.17) | 0.257 |
| **Length of menstrual periods** | 1.08 (0.99-1.17 | 0.102 | 1.06 (0.95-1.18) | 0.324 |
| **Length of menstrual cycle(days)** |  |  |  |  |
| Normal (25-<35) | Ref |  | Ref |  |
| Short (<25 ) | 0.55 (0.22-1.38) | 0.205 | 0.73 (0.29-1.82) | 0.497 |
| Long (≥35 ) | 1.66 (1.10-2.51) | 0.082 | 1.42(0.90-2.23) | 0.127 |
| **Self-reported malaria last 2 months** | 1.72 (1.24-2.38) | 0.001 | 1.33 ( 0.92-1.93) | 0.132 |
| **Malaria at enrolment** | 1.58 (0.93-2.69) | 0.088 | 2.04 (1.15-3.61) | 0.015 |
| **HIV seropositive** | 2.52(1.44-4.40) | 0.006 | 2.57 (1.37-4.81) | 0.003 |
| **Inflammation** | 1.55 (1.04-2.32) | 0.032 | 1.84 (1.18-2.86) | 0.007 |
| **Iron deficiency** | 4.53 (3.77-6.10) | <0.001 | 4.69 (3.45-6.38) | <0.001 |
